# Emergence of differentially regulated pathways associated with the development of regional specificity in chicken skin

**DOI:** 10.1186/s12864-014-1202-9

**Published:** 2015-01-23

**Authors:** Kai-Wei Chang, Nancy A Huang, I-Hsuan Liu, Yi-Hui Wang, Ping Wu, Yen-Tzu Tseng, Michael W Hughes, Ting Xin Jiang, Mong-Hsun Tsai, Chien-Yu Chen, Yen-Jen Oyang, En-Chung Lin, Cheng-Ming Chuong, Shau-Ping Lin

**Affiliations:** Genome and Systems Biology Degree Program, National Taiwan University, Taipei, Taiwan; Genome and Systems Biology Degree Program, Academia Sinica, Taipei, Taiwan; Department of Computer Science and Information Engineering, National Taiwan University, Taipei, Taiwan; Department of Animal Science and Technology, National Taiwan University, Taipei, Taiwan; Research Center for Developmental Biology and Regenerative Medicine, National Taiwan University, Taipei, Taiwan; Department of Pathology, School of Medicine, University of Southern California, Los Angeles, CA USA; Institute of Biotechnology, National Taiwan University, Taipei, Taiwan; International Research Center for Wound Repair and Regeneration, National Cheng-Kung University, Tainan, Taiwan; Department of Bio-Industrial Mechatronics Engineering, National Taiwan University, Taipei, Taiwan; Agricultural Biotechnology Research Centre, Academia Sinica, Taipei, Taiwan; Center for Systems Biology, National Taiwan University, Taipei, Taiwan

**Keywords:** Development, Chicken, Skin, Cosine similarity, Calcium, Histone modifications

## Abstract

**Background:**

Regional specificity allows different skin regions to exhibit different characteristics, enabling complementary functions to make effective use of the integumentary surface. Chickens exhibit a high degree of regional specificity in the skin and can serve as a good model for when and how these regional differences begin to emerge.

**Results:**

We used developing feather and scale regions in embryonic chickens as a model to gauge the differences in their molecular pathways. We employed cosine similarity analysis to identify the differentially regulated and co-regulated genes. We applied low cell techniques for expression validation and chromatin immunoprecipitation (ChIP)-based enhancer identification to overcome limited cell availabilities from embryonic chicken skin.

We identified a specific set of genes demonstrating a high correlation as being differentially expressed during feather and scale development and maturation. Some members of the *WNT*, *TGF-beta/BMP*, and *Notch* family known to be involved in feathering skin differentiation were found to be differentially regulated. Interestingly, we also found genes along calcium channel pathways that are differentially regulated. From the analysis of differentially regulated pathways, we used calcium signaling pathways as an example for further verification. Some voltage-gated calcium channel subunits, particularly *CACNA1D*, are expressed spatio-temporally in the skin epithelium. These calcium signaling pathway members may be involved in developmental decisions, morphogenesis, or epithelial maturation. We further characterized enhancers associated with histone modifications, including H3K4me1, H3K27ac, and H3K27me3, near calcium channel-related genes and identified signature intensive hotspots that may be correlated with certain voltage-gated calcium channel genes.

**Conclusion:**

We demonstrated the applicability of cosine similarity analysis for identifying novel regulatory pathways that are differentially regulated during development. Our study concerning the effects of signaling pathways and histone signatures on enhancers suggests that voltage-gated calcium signaling may be involved in early skin development. This work lays the foundation for studying the roles of these gene pathways and their genomic regulation during the establishment of skin regional specificity.

**Electronic supplementary material:**

The online version of this article (doi:10.1186/s12864-014-1202-9) contains supplementary material, which is available to authorized users.

## Background

The integument system (skin) is essential for an individual to interact with the environment and plays a role in functions such as protection, sensation, thermoregulation, camouflage, and mating. These functions are achieved through evolutionally specialized skin and its appendages located in a regional-specific manner. In humans, these regions include the scalp, face, eyebrow, and sweat glands for communication and metabolic homeostasis. In birds, feathers provide thermal insulation, protection, communication, and the ability to fly. To form these specialized skin ectodermal organs during development, the ectoderm gradually becomes diversified through interactions with the dermis [1]. How this process occurs at the genomic level is poorly understood. Among experimental animals, avian integuments provide an ideal model with which to address this question.

Avian species evolved feather-forming regions on the body and scale-forming regions on the feet that represent distinct skin morphologies and appendages. The feather-growing skin developed patterns that provide optimal feather coverage while maintaining strong integrity of each feather [2]. On the other hand, avian foot skin developed a hardy scale surface to provide strong protection against rough ground surfaces and harsh environments [2]. Such skin specialization is a fundamental morphogenetic process that facilitates the function of different body regions of an individual.

Feather skin development follows sequential processes starting from placode formation, dermal papilla assortment, formation of a WNT gradient, and finally to region-specific appendage formation [3-5]. Some adhesion molecules and signaling molecules are expressed homogeneously and are gradually restricted to either bud or inter-bud regions (restrictive mode); other regulatory elements are expressed *de novo* (*de novo* mode) [6]. For example, *FGF10* expression initiates feather placode formation prior to the expression of feather bud-suppressive *BMP2* [7]; *SHH* expression promotes feather bud development; *BMP2* and *BMP4* modulate feather track size by suppressing feather bud fate [8,9]; and the *WNT/beta-catenin* signaling pathways were found to be involved throughout skin morphogenesis from feather bud formation to topological arrangement [3,10,11]. These signaling pathways either activate or inhibit bud formation and systematically regulate fate decision for skin appendages. The dynamic equilibrium of the cell number and activator/inhibitor ratio give rise to the final skin pattern [12].

Despite diverse fate decisions for feather/scale bud development, these patterning units share moderate similarity in gene expression patterns, such as posterior *SHH* and anterior *BMP2* expressions in the bud [13]. Several WNT family members, such as *WNT1*, *3a*, *5a*, *6*, and *11*, were found to have specific expression sites that contribute to the shaping of the feather bud during feather skin development [3,11]. Skin reconstitution assays demonstrated that completely dissociated embryonic skin epidermis cells can reform periodic patterns without reference to previous positional codes [6]. These findings provided strong evidence that skin pattern and morphogenesis are predetermined and are tightly regulated.

Interestingly, classical studies have shown that retinoic acid treatment is self-sufficient for inducing feather formation in the tarsometatarsal scale region [14]. Other feather-developing genes, such as *Delta1* and *beta-catenin*, were also found to be self-sufficient for inducing feather development on scale skin [15,16]. The suppression of feather bud-suppressive BMP signaling by the dominant-negative *BMP* receptor converts scales into feathers [17]. On the other hand, elevated *BMP12* expression was found to cause the naked neck trait in domestic fowl [18]. Ectopic expression of these causal regulators has demonstrated that a fairly simple genetic switch can shift the state of skin development from feather to scale skin or vice versa.

Although embryonic skin demonstrates the characteristics of being dynamic and self-organizing, which give rise to skin patterns, early skin epithelium (E7 feather and E9 scale) is highly plastic for differentiation into either feather or scale-like patterns [19-21]. The morphogenic plasticity of skin epithelium declines it gains regional specificity. This phenomenon implies the presence of paracrine positive feedback loops between the epithelium and mesenchyme. Recombination experiments further demonstrated that the feather/scale patterning on skin epidermis is highly dependent on its dermal partner [19,21], suggesting that developmental decisions may be the result of differentially controlled dermal signaling. However, differences in the molecular controls between early embryonic feather and scale skin specification are not fully understood. Genome-wide RNA expression analysis joining the comparisons of feather and scale skins, before and after skin differentiation, and epithelia and mesenchyme can provide a novel perspective to our understanding of the regional specificity determination of the skin.

Using the feather/scale as a model, we aspire to use bioinformatics methodology for the identification of novel regulatory pathways that are potentially involved in the feather/scale developmental decision. Specifically, we adapted the cosine similarity analysis to identify gene groups that show regulatory concordance (see Methods) during early skin development. Some of these variable gene expression levels are likely associated with epigenetic regulatory mechanisms in addition to signaling transduction cascades. To evaluate this level of regulation, we characterized candidate enhancer regions with differential chromatin modifications on several selected genes and confirmed the validity of this approach. This work shows several pathways that may be involved in the fate decision or differentiation of different skin regions. Future investigation will elucidate the functions of these pathways and how different skin regions are established.

## Methods

### Animal ethics statement

The fertilized eggs used in this study were incubated in the egg incubators in Institute of Biotechnology at National Taiwan University, and in Department of Pathology, School of Medicine at University of South California. The animal use protocol and dissection techniques [21] have been reviewed and approved by the Institutional Animal Care and Use Committee in National Taiwan University and in University of South California. All efforts were made to minimize suffering.

### Microarray Profiles

Chicken embryonic skin microarray profiles, which had been described by Hughes et al., were obtained from Chuong’s laboratory [21]. The profiles were obtained from the feather-forming dorsal and scale-forming metatarsal skin compartments, including two E7 dorsal skin epithelial (E7fe), three E7 dorsal skin mesenchymal (E7fm), two E9 dorsal skin epithelial (E9fe), three E9 dorsal skin mesenchymal (E9fm), two E9 metatarsal skin epithelial (E9se), three E9 metatarsal skin mesenchymal (E9sm), two E11 metatarsal skin epithelial (E11se), and three E11 metatarsal skin mesenchymal (E11sm) compartments [21]. The expression profiles of the 20 microarray assays were combined into a common set of known chicken genes. Raw array data were normalized using the Robust Multi-array Average technique. The array-specific bias was eliminated through quantile normalization (R: affy package) [22,23]. The microarray profiles have been deposited in Gene Expression Omnibus (GEO) data repository under accession number GSE62882.

### Cosine Similarity Analysis

The property of cosines can be used to evaluate the similarity between trends of data progression and has been widely adapted in data mining and clustering strategies [24-26]. With the 20 microarray data points we generated from various regional and temporal embryonic chicken skin samples, we gathered the 20-dimensional data points for each gene, in which the *i*^th^ dimension represents the gene’s expression in the *i*^th^ microarray, where *i* denotes the 1^st^ to 20^th^ microarray profiles. The regulatory concordance of any 2 genes can be presented by a cosine value from 1 to −1 corresponding to 0° to 180° angles. When genes are co-regulated, the cosine value approximates to 1. Genes that are reciprocally regulated have a cosine value approximates to −1. A cosine value close to 0 suggests a pair of genes have little regulatory concordance.

**Figure 1:**
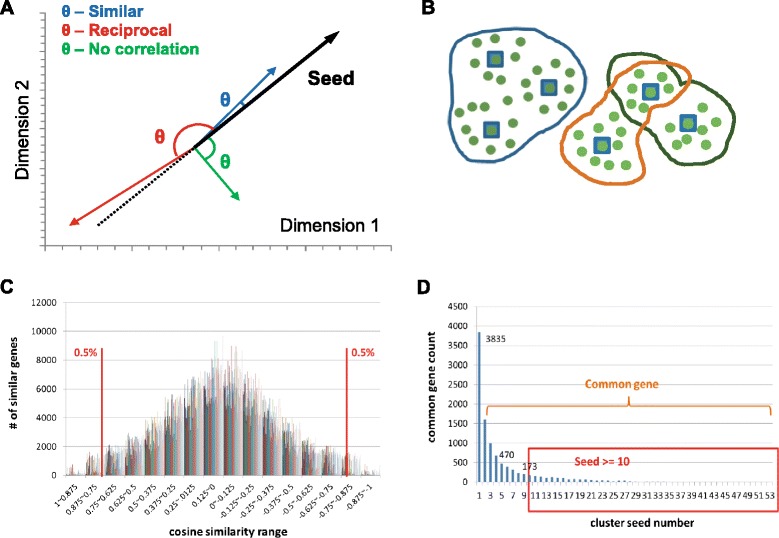
**Cosine similarity analysis methodology for determining co-differentially regulated genes in the feather/scale region. A)** 2D representation of the concept of cosine similarity analysis. The actual analysis occurs in a 20-dimension hyperspace. θ represents the angles between a probe vector and a differentially expressed vector. **B)** 2D representation of the clustering concept after the identification of similarly regulated vectors. Differentially expressed genes and their correlated probes are represented by blue squares and green circles, respectively. The correlated differentially expressed genes and probes are clustered (encircled gene sets by the outlines of different colors representing different clusters). Some differentially expressed genes and probes may be clustered into more than one group. **C)** Demonstration of cosine value distribution for each seed. The cosine value distributions are normalized by Fisher transformation. The upper 0.5% and lower 0.5% mark the selection boundaries in choosing probes with similar regulation patterns. **D)** Distribution of genes that share common seeds. Genes sharing more than 10 common seeds are shortlisted for their potential roles in developmentally important pathways.

For two data points *a* and *b*, representing the expression values of gene *a* and *b* in the 20 microarrays, their inner product space measured by the cosine function is defined as follows: $cos\theta =\frac{a\cdot b}{\left|a\right|\left|b\right|}=\frac{\sum_k{a}_k{b}_k}{\sqrt{\sum_k{a}_k^2{\sum}_k{b}_k^2}}$, where θ is the angle between *a* and *b*, illustrating the difference of their regulatory trends [27]. Genes that show regulatory concordance are clustered (Figure 1A, B).

#### Seed (candidate genes) selection

We defined differentially expressed genes as “seeds” if they have greater than 5-fold expression differences between feather- and scale-forming skin tissues. Specifically, we selected seeds by performing the following 4 groups of comparisons between feather- and scale-forming skins of similar developmental plasticity: E7fe vs. E9se, E7fm vs. E9sm, E9fe vs. E11se, and E9fm vs. E11sm (E7, E9, and E11 embryonic days, feather (f; dorsal) and scale (s; metatarsal) skin region, and epithelium (e) and mesenchyme (m) skin compartments follow the descriptions by Hughes et al. [21]).

#### Identification of genes co-regulated or reciprocally regulated with seeds

We calculated the spectrum of cosine values by comparing each gene to a particular seed and divided the resulting cosine value distribution into 16 even sections from −1 to 1. We applied Fisher Transformation (R: psych package) [22,28] to normalize the cosine value distribution for each of the 4 groups of seeds (E7fe/E9se; E7fm/E9sm; E9fe/E11se, E9fm/E11sm; Figure 1C). Genes in the top 0.5% and bottom 0.5% of the cosine value distribution are defined as co- and reciprocally expressed genes to a seed. With total of 1% of the whole microarray probe set, we ensured that the probes/genes selected have very similar or reciprocal expression to the corresponding seeds.

#### Identification of key regulators by exploratory data analysis

The exploratory data analysis approach is suitable for preliminary discoveries of data structure and checking whether the data are consistent with the study expectations [27]. First, we grouped seeds with genes that share regulatory concordances. We then clustered these groups according to whether their seeds are co- or reciprocally regulated (Figure 1B). Some genes may be assigned to multiple gene clusters. We deemed that a gene shared by multiple clusters may play potential roles in the regulatory crosstalk among these clusters. Genes showing a strong correlation (similar or reciprocal) with more than 10 probes were enlisted as genes of interest being co- or differentially regulated between feather and scale skins (Figure 1D).

### Sample Collection for Gene Expression and Chromatin Analysis

We collected 8 sets of embryonic chicken (Cobb500 Broiler) skins from both feather and scale regions that represent predicted plastic and differentiated states: E7fe, E7fm, E9fe, E9fm, E9se, E9sm, E11se, and E11sm. E7, E9, and E11 are the egg incubation days and reflect Hamburger–Hamilton stages (HH) [29] of HH30, HH34, and HH37, respectively. The feather epithelium (fe), feather mesenchyme (fm), scale epithelium (se), and scale mesenchyme (sm) were collected separately. For separation of the skin epithelium and mesenchyme, skins were treated with 2× HBSS buffer at 4°C for 5 min prior to fine forceps separation.

### RNA Purification

Approximately 10–20 mg of skin samples from 1–4 embryos were homogenized in 1 ml of TRIzol® reagent (15596–018; Invitrogen) for each biological repeat. Two hundred microliters of chloroform was added, followed by phase separation by centrifugation at 12,000 g for 15 min at 4°C. To isolate RNA, the supernatant aqueous phase was aspirated and mixed with 1 volume of isopropanol. The RNA was pelleted by centrifugation at 12,000 g for 10 min at 4°C. RNA pellets were washed with 75% ethanol and resuspended in pure water.

### Reverse Transcription-qPCR (RT-qPCR)

Reverse transcription was performed using the Invitrogen™ SuperScript® III First-Strand Synthesis System according to the RT-PCR manual. Briefly, each 20 μl reaction contained the following: 1 μg of purified RNA, 50 ng of random hexamers, 1 μl of 10 mM dNTPs, and 10 μl of cDNA Synthesis Mix. Samples were incubated using the following PCR parameters: 10 min at 25°C, 50 min at 50°C, and 5 min at 70°C. Next, the samples were chilled on ice and then stored at −30°C for future use. Real-time PCR was performed using a Roche Light Cycler® 480II and the SYBR Green PCR Master Mix. The RT-qPCR primers used are listed in Additional file 1: Table S1. *GAPDH* and *beta-actin* were used to normalize gene expression for cytosolic proteins and membrane proteins, respectively.

### In Situ Hybridization

To make the antisense probe for in situ hybridization, two PCRs were performed for each gene. In the first PCR, we used the primer set Forward1 + Backward1. cDNA from E7 skin was used as template. The single band PCR product was purified with a gel purification kit (Qiagen). In the second PCR, we used the primer set Forward1 + the nested primer Backward2-T7. The previously purified PCR product was diluted 1:100 and used as the template. For CACNA1D, Forward1: CAG CAA TAT GCC AAG AGC AA; Backward1: TAG ATC CAT GGC CAT TCA CA; Backward2-T7: CTA ATA CGA CTC ACT ATA GGG ACT GAC GTC CAT TCC CTG AG. For CACNA2D1, Forward1: ACG GCA AAC GTC TTA CCA AC; Backward1: TTC ACA GGC CAT CCA CTG TA; Backward2-T7: CTA ATA CGA CTC ACT ATA GGG TCA CCA CCA TCC GTA AAC AA. The PCR product was gel purified and sequenced with T7 primer to verify its identity. An antisense RNA probe was generated using DIG RNA Labeling Kit (Roche). Whole mount in situ hybridization was performed according to Jiang et al. [30]. For each probe, the E7 and E9 samples are kept in the same tube to perform in situ hybridization and develop color over the same time period. One set of samples without probe added was used as the control. Photos for different samples are taken using similar lighting conditions.

### Chromatin Immunoprecipitation (ChIP)-next generation sequencing (ChIP-seq) and qPCR

Skin epithelium samples from 1–4 embryos per biological repeat were treated with 0.0625% trypsin and 20 μM collagenase IV for 2 min at 37°C for homogenization, and this reaction was stopped with fetal bovine serum (10100147; Gibco®). The homogenized samples were fixed with 1% formaldehyde at room temperature and stopped by 125 mM glycine. Cell nuclei were extracted with 0.5% NP-40 and 0.25% Triton X-100. Formaldehyde-fixed nuclei were resolved and sonicated following Diagenode’s® Low Cell# ChIP manual (kch-maglow-G48; Diagenode) to overcome the limited cell availability from embryonic skin epithelium. The following antibodies were used for the immunoprecipitation: anti-H3K4me1 (ab8895; Abcam), anti-H3K27ac (ab4729; Abcam), and anti-H3K27me3 (#07-449; Millipore). Antibodies were pre-bound with protein G-coated magnetic beads for 2 hours in 4°C. A total of 1 × 10^5^ cells was used for each 100 μl ChIP reaction overnight at 4°C. ChIP samples were collected following the manufacturer’s instructions. ChIP and input libraries were prepared according to the Illumina protocol and sequenced using an Illumina HiSeq™ Sequencing System. Sequencing reads were trimmed using CLC Genomics Workbench v6.0.2 (CLC Bio, Aarhus, Denmark), mapped to chicken genome assembly galGal4 with Bowtie2 v2.1.0 [31], and analyzed with MACS v1.4.2 [32] for peak calling using the default settings. Real-time PCR was carried out on a Roche Light Cycler® 480II using the SYBR Green PCR master mix. As the loading control, 10% input was used to normalize the recovery rate in each sample of the same ChIP antibody. The primers used were designed according to our unpublished ChIP-seq dataset and are listed in Additional file 1: Table S2.

## Results and discussion

### Identification of differentially regulated and co-regulated pathways in different skin regions using cosine similarity analysis

**Figure 2:**
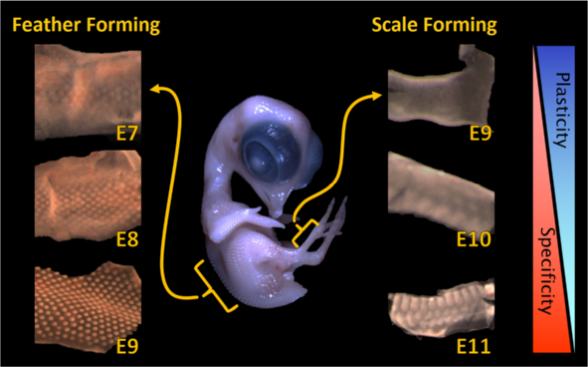
**Developmental progress of feather skin and scale skin.** Left side of chicken embryo: feather skin development from E7 to E9. Right side of chicken embryo: scale skin development from E9 to E11. The development stages for feather and scale skins are matched; top to down represents the developmental trend from undifferentiated skins to differentiated skins. During the process, skin gradually loses developmental plasticity in exchange for gaining regional specificity (right panel).

Feather and scale skin development is a systematic event and involves dynamic regulation to gain regional specificity. Because feather skin development (E7) starts earlier than scale skin development (E9), comparisons are feasible only by matching feather and scale developmental stages using embryos of different ages (Figure 2) [19]. To identify known regulatory pathways that are not yet known to be involved in feather/scale development and fate decision, we applied Gene Set Enrichment Analysis (GSEA) [33,34] to compare skins of similar developmental competence: E7 feather vs. E9 scale and E9 feather vs. E11 scale (Additional file 1: Figure S1). We also applied GSEA for the regulatory network changes associated with development using comparisons between a competent skin against its differentiated counterpart — E7 feather vs. E9 feather; E9 scale vs. E11 scale. We found that most of the developmentally associated genes identified from GSEA were involved in regulatory networks, such as BMP-SHH, WNT, Delta-Notch, and retinoic acid pathways, which have been well described in regional skin development [12,14-17,35,36] (Additional file 1: Figure S1).

Next, we sought to identify novel regulatory factors or pathways important for skin feather/scale fate decision. Because genes along a regulatory pathway share either similar or reciprocal regulation patterns under this developmental context [37-39], we considered cosine similarity analysis to be a potentially valid approach because this method presented straightforward identification of co- or reciprocally regulated genes (1 or −1, respectively). We measured the expression correlations between genes that were differentially expressed in feathers and scales (E7fe vs. E9fe, E7fm vs. E9fm, E9se vs. E11se, and E9sm vs. E11sm) [21]. In each comparison, a set of differentially expressed probes with fold changes greater than 5 was selected. Cosine values of each probe against each differentially expressed probe were calculated. The discrete cosine value distributions at 0.125 intervals were observed to approximate a normal distribution (Figure 1C). Next, we standardized the cosine value distributions through Fisher’s transformation [40] and then defined the top 0.5% of genes with cosine values close to 1 or −1 in a distribution as potential co- or reciprocally regulated genes. To reduce false positives, we filtered the probes for those correlated with more than 10 differentially expressed probes (Table 1). The differentially expressed/co-regulated genes that were identified from these comparisons (Additional file 1: Table S4) were utilized for analysis using the STRING database (http://string-db.org/) [41], in which the embedded Hypergeometric test [42] were used for evaluating the statistical significance of these genes. Genes in the co-regulated/differentially expressed list were compared with the KEGG database. We identified ECM-receptor interaction, focal adhesion, melanogenesis, calcium signaling pathway, and vascular smooth muscle contraction as the top 5 pathways in which most of the differentially expressed candidates were substantially involved (Table 2). The WNT signaling pathway is also one of these regulated pathways identified from our cosine similarity analysis. Because the importance of WNT pathway in feather differentiation has been clearly demonstrated, the identification of this pathway through our cosine-similarity assay gave us confidence that other novel pathways we identified may also play key roles in feather/scale differentiation.

**Table 1:** **Candidate microarray probes from the cosine similarity analysis**

**Comparison**	**Differentially expressed probes**	**Co-regulated probes**	**Co-regulated probes correlated with ≥ 10 seeds**
E7fe vs. E9se	127	8,835	1,592
E7fm vs. E9sm	151	8,642	2,019
E9fe vs. E11se	131	8,796	1,740
E9fm vs. E11sm	124	8,123	1,955

Numbers of microarray probes that are differentially regulated (seeds), co-regulated with seeds, and co-regulated probes sharing expression patterns with more than 10 seeds are shown.

**Table 2:** **Top 5 significant KEGG pathways identified based on the differentially regulated genes and co-regulated genes from cosine similarity analysis**

**KEGG Pathways**	**E7fe vs. E9se**	**E7fm vs. E9sm**	**E9fe vs. E11se**	**E9fm vs. E11sm**
ECM-receptor interaction	4.94e-08	1.59e-03	5.78e-09	2.06e-03
Focal adhesion	3.88e-08	1.39e-05	1.32e-07	6.21e-04
Melanogenesis	3.34e-05	2.89e-02	2.29e-07	1.46e-02
Calcium signaling pathway	5.79e-03	5.28e-05	2.23e-02	>0.05
Vascular smooth muscle contraction	>0.05	2.30e-05	>0.05	7.21e-04

Differentially expressed genes and co-regulated genes from each feather-scale comparison with similar developmental plasticity. KEGG pathways are ranked based on the number of comparisons showing high significance (p-value < 0.05).

From the top-listed KEGG regulatory pathways, a rich set of intercellular interactions found in the ECM-receptor interaction and focal adhesion suggested significant skin region-specific expressions of skin membrane proteins. The ECM interactions and subsequent molecular cascades may lead to focal adhesion activities. Previous studies on the pattern formation of early feather skin reported that several families of cell adhesion molecules may play a central role in pattern formation [10,43]. The cell adhesion activities followed restrictive modes and drove the formation of cell aggregates (10–25 cells) in the skin dermal region [44]. The stabilized cell aggregates underwent dermal condensation and acquired the ability (inducer) to send dermal messages to initiate (induce) epidermal placode formation [45-47]. Our findings of laminin, fibronectin, and other adhesion protein members within the differentially expressed genes in our pathway analysis supported the concept that specific intercellular communication could direct integument pattern formation. However, ECM interaction and focal adhesion pathways lead to divergent intercellular interaction and a broad spectrum of downstream responses, making it difficult to trace specific molecular interaction cascades and downstream activation targets.

### The calcium signaling pathway is differentially expressed in developing feather and scale regions

**Figure 3:**
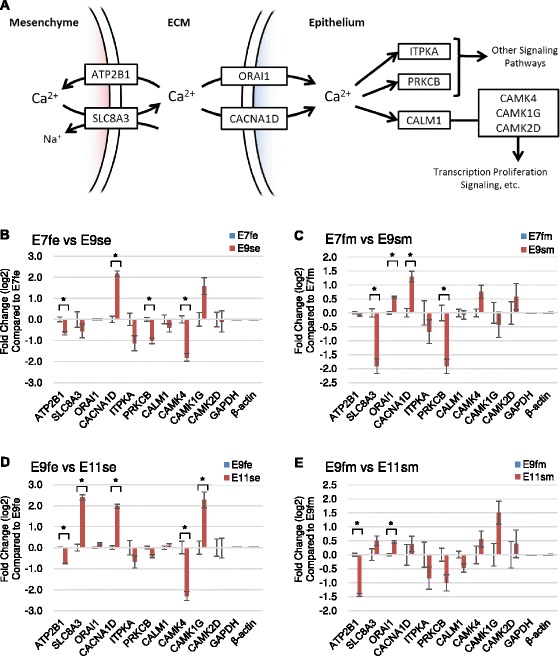
**Differentially regulated genes involved in calcium signaling pathways. A)** Genes along the calcium signaling pathway that may be differentially regulated in the epithelium and mesenchyme. Only differentially expressed genes and co-regulated genes identified by cosine similarity analysis are shown. **B-E)** Expression comparisons of calcium signaling pathway-associated, differentially expressed genes and coregulated genes between feather and scale skins of similar plasticity: **B)** E7fe vs. E9se; **C)** E7fm vs. E9sm; **D)** E9fe vs. E11se; and **E)** E9fm vs. E11sm. *represents p-values < 0.05 for the bracketed comparisons; biological repeats ≥ 3.

Given that the differentiation of embryonic skin epithelium is driven by mesenchymal signaling, interactions between the two skin compartments likely play major roles in modulating epithelial gene expression. Among the identified top pathway candidates (Table 2), the calcium signaling pathway demonstrated regulatory preferences on scale-forming epithelium. The up-regulated genes were found along the mesenchymal calcium export pathway, epithelial calcium import pathway, and pathways that lead to transcriptional regulation (Figure 3A). Next, we performed RT-qPCR to validate the differentially expressed genes in the calcium signaling pathway during feather and scale development (Figure 3B-E). For the analysis of skin epithelial responses to mesenchymal signaling, we identified a five-fold elevated expression of *CACNA1D* and *CAMK1G* in early scale-forming epithelium compared with the feather-forming counterpart (Figure 3B). In contrast, the ligand-gated calcium channel ORAI1 identified from cosine similarity analysis did not show significant expression differences across all embryonic epithelial skin regions. For the early feather mesenchyme, *SLC8A3* and *PRKCB* (Figure 3C) demonstrated higher expression than the early skin mesenchyme. These data suggested the region-specific expression of members of the calcium signaling channel gene family.

Although the role of calcium signaling in the scale-feather switch remains elusive, several downstream effectors of calcium signaling play important roles in epidermal differentiation. Protein kinase C (PKC) is one of the important transactivating effector pathways in response to calcium. Several isoforms of PKC contribute to various events in epidermal development in response to calcium-phospholipase C signaling. For example, PRKCB stimulates melanogenesis by activating tyrosinase [48]. Accordingly, we detected lower *PRKCB* in the scale-forming epidermis, particularly in the differentiating stage, than in the feather-forming scales of hens, whose legs are less pigmented compared with the colored feather coat on the body. Another important effector pathway is modulated by calmodulin (CaM), which can activate CaM kinases (CaMKs) upon binding to calcium. Calcineurin is one of the key targets of CaM-CaMK signaling, and it plays a key role in governing the proliferation and differentiation of keratinocytes together with NFAT and Notch [49]. Interestingly, a diverse response might be elicited by different CaMKs, such as non-canonical Wnt/Ca^2+^ signaling through WNT5a and CaMK2 for ventral cell fate decision [50]. Our results showed increased CaMK1 and decreased CaMK2 and CaMK4 in scale-forming tissue, implying divergent developmental processes governed by different sets of CaMKs.

**Figure 4:**
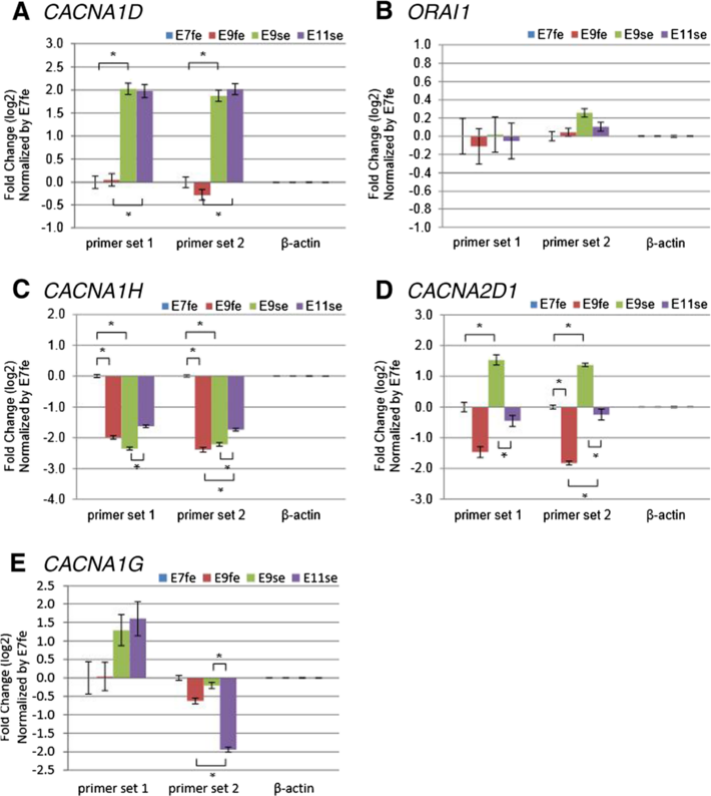
**RT-qPCR validation of the differentially regulated calcium channel genes at the epithelium (biological repeats ≥ 3).** Cosine similarity-identified **A)** *CACNA1D* and **B)** *ORAI1* were tested with different primer sets. Other high voltage-gated calcium channels **C)** *CACNA1H* **D)** *CACNA2D1* and **E)** *CACNA1G* showed distinct expression patterns between feather and scale epithelium. *represents p-values < 0.05 for the bracketed comparisons.

**Figure 5:**
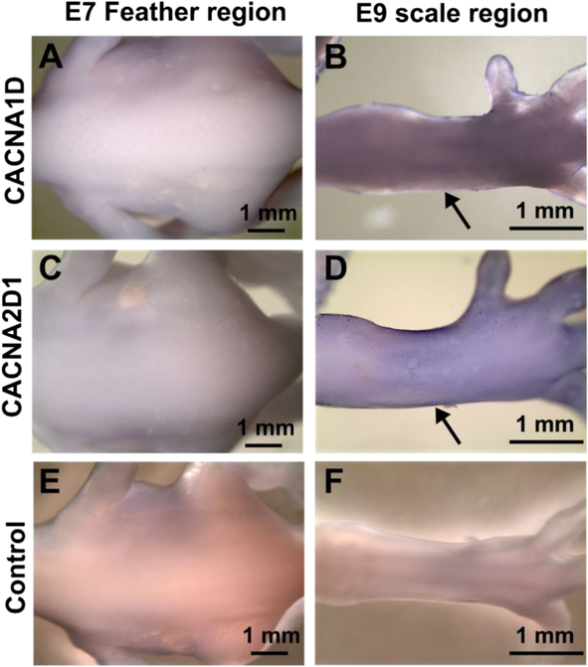
**Whole mount in situ hybridization of CACNA1D and CACNA2D1 in E7 feather forming and E9 scale forming regions. A, B)** CACNA1D; **C, D)** CACNA2D1; **E, F)** control; **A, C, E)** E7 feather forming region; **B, D, F)** E9 scale forming region. Arrows in B and D indicate higher expression levels in the scale forming regions.

Calcium signaling pathways are known to be involved in the regulation of human skin development, particularly in keratinocyte development [51,52]. Nevertheless, calcium signaling pathways have not yet been shown to be differentially regulated during early feather/scale skin development. Because calcium signaling is initiated by calcium ion influx, we further investigated the expression of calcium channel subunits in the skin epithelium (Figure 4), as these units show high developmental plasticity and elevated calcium channel expression at early stages. As expected, *CACNA1D* expression is stably up-regulated in the scale skin epithelium by 4-fold compared with that in the feather skin epithelium (Figure 4A). Comparatively, the ligand-gated *ORAI1* showed almost no expression variation in any of the tested embryonic skin epithelium samples (Figure 4B). We also tested other voltage-gated calcium channel α_1_ and α_2_δ subunits (Additional file 1: Table S2 and Additional file 1: Figure S2). We specifically picked these genes because their expressions are not limited to neuronal lineages. Among the tested calcium channel subunits, we observed a transient up-regulation of *CACNA1H* in early feather skin epithelium by approximately 4-fold compared with other tested skin epithelium (Figure 4C). We also observed a moderate *CACNA1H* up-regulation in E11se compared with E9se. Conversely, *CACNA2D1* expression seemed to be favored at earlier developmental stages (Figure 4D), although the level of expression may differ between the feather and scale epithelium. The distinct combination of calcium channel expression may be spatio-temporally regulated to ensure proper levels of calcium-dependent activities for feather/scale skin development and differentiation. Whole-mount in situ hybridization on *CACNA1D* and *CACNA2D1* supported their expressions favored in early metatarsal scale skins compare to early dorsal feather skins (Figure 5). We observed no obvious geometric patterns for the strong expression of these calcium channel subunits in the scale-forming region (Figure 5B,D), which may correspond with the fact that the scale patterning is not clearly identifiable in E9 metatarsal skins [29,53]. Interestingly, CACNA1D showed faint expression coincide with the feather bud region (Figure 5A) and may imply its potential involvement in early feather bud development. The complexity of the expression regulation of the voltage-gated calcium channels supported the notion that calcium is involved in differentially regulated pathways that lead to the development of feather and scale skins. Additionally, expression discrepancies between calcium channels may indicate potential sites of alternative transcription in a tissue-specific manner.

### Potential enhancers for calcium channel genes are identified based on the specific combination of histone modifications

**Figure 6:**
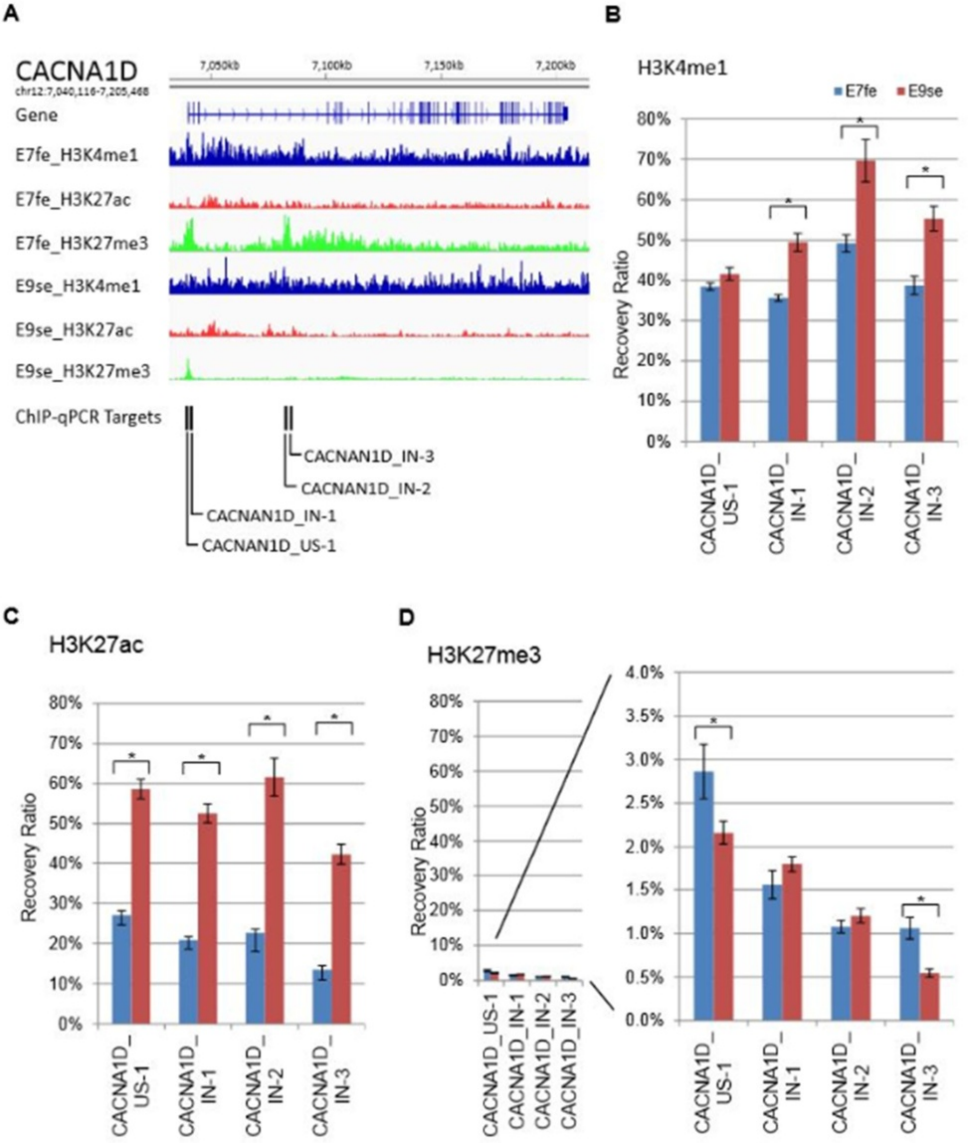
**ChIP-qPCR of the potential enhancer regions that may be associated with** ***CACNA1D*** **gene activities. A)** Mapped ChIP-seq reads on and near *CACNA1D*; ChIP-seq on histone markers H3K4me1, H3K27ac, and H3K27me3 was performed for undifferentiated feather epithelium from embryonic day 7 (E7fe) and undifferentiated scale epithelium from embryonic day 9 (E9se). The positions chosen for ChIP-qPCR amplifications (black vertical bars) were designed based on the most differentially marked regions identified by MACS peak calling analysis. **B-D)** ChIP-qPCR results for enhancer-associated histone markers: H3K4me1, H3K27ac, and H3K27me3, respectively. Results are arranged in sequence from left to right, matching the sites for ChIP-qPCR amplification in **A)**. “US” denotes primer set targeting the region upstream of the gene; “IN” denotes primer set targeting the intron of the gene. *represents p-values < 0.05 for the bracketed comparisons.

Because early embryonic skin epithelium possesses regional developmental plasticity for feather and scale lineages, the correlative expression patterns of the calcium signaling pathway genes may be involved in the developmental process. These spatio-temporal-specific expression patterns are very likely regulated in association with histone modifications on gene enhancers. Histone modification is highly associated with many biological traits such as developmental states, cell type, and tissue type. Because very few enhancer regions have been identified in the chicken model, and enhancer predictions based on sequence conservation may be limited, constructing genome-wide histone modification profiles is of interest. Utilizing the patterns of histone signatures (H3K4me1, H3K27ac, H3K27me3, H3K4me3 and p300 activity) in enhancer regions in embryonic stem cell differentiation from the literature [54], we applied chromatin immunoprecipitation (ChIP) and next-generation sequencing (ChIP-seq) to construct profiles focusing on early skin epithelium (E7fe and E9se) (data not shown). The experiment was conducted using limited cell numbers (10^5^ cells) due to low cell availabilities in embryonic chicken skin tissue, particularly the skin epithelium during plastic stages (E7fe and E9se). Based on our preliminary peak calling analysis results, we designed ChIP-qPCR primers targeting these potential enhancer sites near calcium channel genes (Figure 6, Additional file 1: Table S2, Additional file 1: Figure S3–7). For CACNA1D, three peaks at the approximate transcription start site (TSS) showed increased H3K27ac at E9se, and increased H3K27me3 at E7fe. Similar peaks were found in intron 3 of the gene (Figure 6). These findings supported higher *CACNA1D* expression at E9se than that at E7fe. Comparatively, *CACNA1H* showed moderately higher H3K27ac and lower H3K27me3 recovery at the tested ChIP peaks at E7fe than at E9se. The signatures corresponded with higher *CACNA1H* expression for early feather epithelium E7fe, as expected (Additional file 1: Figure S3). For *CACNA2D1*, a regulatory hotspot found at intron 2 also showed increased H3K27ac at E9se, but less significant changes were observed in the H3K27me3 levels (Additional file 1: Figure S4). These histone signatures correlated well with the expression profiles found at *CACNA1D* and *CACNA2D1,* respectively. These results support the idea that histone modification may regulate the associated enhancer activities in scale- and feather-forming skin [55]. Moreover, studies have demonstrated that calcium influx through L-type voltage-sensitive calcium channels was found to induce enhancer-associated CBP/p300 activities [56] and facilitate histone H3 acetylation through PKCγ phosphorylation [57]. Such a mechanism likely introduces activation signatures to the target genes. The tissue-specific histone modification may result in a positive feedback regulation that increases calcium concentration. Particularly, *CACNA1D* showed transcriptional preferences on scale-forming skin and presented histone signatures that correlated with high transcription at multiple regulatory hotspots for scale-forming skin. Calcium, in general, is required to initiate the differentiation of keratinocytes [51,58]. The accumulation of extracellular calcium was found to be involved in the left-right asymmetric activation of Notch during gastrulation of the chicken embryo [59]. Moreover, a calcium signaling mediator, calmodulin, was found to be involved in avian beak length [60]. These lines of evidence suggest that calcium signaling may play important roles in differentiation and regional specialization. The differentially regulated calcium channel genes we observed in embryonic skin also support the roles of calcium-dependent differentiation signaling pathways. Regarding scale-forming epithelium, the trend of high expression levels of calcium channels may likely increase intracellular calcium, leading to an alternative route to the WNT signaling pathway that bypasses β-catenin [61], to the reduction of β-catenin activity [62,63] and to the demotion of β-catenin-associated feather bud formation [10]. Such speculation poses an interesting line of study for further analysis of calcium channel activities in the regulation of embryonic skin development.

## Conclusion

In summary, we demonstrated the applicability of cosine similarities in genome-wide studies for the identification of novel regulatory pathways associated with the differential developmental pathways of embryonic feather and scale skins. We found that calcium signaling pathways are involved in feather/scale developmental bifurcation at the expression level. We demonstrated that voltage-gated calcium channels are expressed spatio-temporally. In particular, *CACNA1D* showed expression preferences toward scale-forming embryonic epithelium. Furthermore, we showed potential correlations between the expression of voltage-gated calcium channels and their histone signatures that are associated with potential enhancers via H3K4me1, H3K27ac, and H3K27me3 marks. Similar tissue specificities were found for *CACNA2D1* and reciprocal for *CACNA1H*. Thus, we showed that the tissue-specific association of voltage-gated calcium channels mediated calcium signaling pathways with the development of feather- and scale-forming embryonic chicken skin. These calcium channels may be used in regulating early feather and scale development or for distinct differentiation pathways of feather/scale phenotypes. Additional functional experiments are required to characterize the roles of calcium channels during avian skin development. Nevertheless, the co-regulated differentially expressed genes described here present novel opportunities for exploration.

## Additional file

Additional file 1: Table S1. Primer sets for RT-qPCR. **Table S2.** Primer sets for ChIP-qPCR. **Table S3.** KEGG pathways identified based on the differentially regulate genes and co-regulated genes from cosine similarity analysis. **Table S4.** Cosine similarity analysis results, for E7fe vs E9se; E7fm vs E9sm; E9fe vs E11se; E9fm vs E11sm. **Figure S1.** GSEA coupled with interaction pathway analysis on embryonic development GO terms for the comparisons between feather and scale of matching developmental stages (E7fe vs E9se, E7fm vs E9sm, E9fe vs E11se, and E9fm vs E11sm); as well as for the comparison of same skin component before and after development (E7fe vs E9fe, E7fm vs E9fm, E9fe vs E11fe, and E9fm vs E11fm). **Figure S2.** RT-qPCR of calcium channel members at the epithelium (biological repeats ≥ 3). **Figure S3.** ChIP-qPCR of the potential enhancer regions that may be associated with *CACNA1H* gene activities. **Figure S4.** ChIP-qPCR of the potential enhancer regions that may be associated with *CACNA2D1* gene activities. **Figure S5.** ChIP-qPCR of the potential enhancer regions that may be associated with *CACNA1C* gene activities. **Figure S6.** ChIP-qPCR of the potential enhancer regions that may be associated with *CACNA1G* gene activities. **Figure S7.** ChIP-qPCR of the potential enhancer regions that may be associated with *CACNA2D3* gene activities.

## Abbreviations

ChIP: Chromatin immunoprecipitation
ChIP-seq: ChIP next-generation sequencing
E7fe: Embryonic day 7 feather-growing skin (dorsal) epithelium
E7fm: Embryonic day 7 feather-growing skin (dorsal) mesenchyme
E9fe: Embryonic day 9 feather-growing skin (dorsal) epithelium
E9fm: Embryonic day 9 feather-growing skin (dorsal) mesenchyme
E9se: Embryonic day 9 scale-growing skin (metatarsal) epithelium
E9sm: Embryonic day 9 scale-growing skin (metatarsal) mesenchyme
E11se: Embryonic day 11 scale-growing skin (metatarsal) epithelium
E11sm: Embryonic day 11 scale-growing skin (metatarsal) mesenchyme
FC: Fold change
RT-qPCR: Reverse transcription-real-time polymerase chain reaction
GSEA: Gene set enrichment analysis
STRING: Search tool for the retrieval of interacting genes/proteins
KEGG: Kyoto encyclopedia of genes and genomes
ECM: Extracellular matrix
TSS: Transcription start site
PKC: Protein kinase C
PRKCB: Protein kinase C, beta
PKCγ: Protein kinase C, gamma
CaMK: CaM kinase
WNT: Wingless-type MMTV integration site family
BMP: Bone morphogenetic protein
SHH: Sonic hedgehog
FGF: Fibroblast growth factors
CACNA1D: Calcium channel, voltage-dependent, L type, alpha 1D
CAMK1G: Calcium/calmodulin-dependent protein kinase I gamma
SLC8A3: Solute carrier family 8 (sodium/calcium exchanger), member 3
ORAI1: ORAI calcium release-activated calcium modulator 1
H3K4me1: Histone H3, Lysine 4 mono-methylation
H3K27ac: Histone H3, Lysine 27 acetylation
H3K27me3: Histone H3, Lysine 27 tri-methylation
