# Supplementary material for: Emergence of differentially regulated pathways associated with the development of regional specificity in chicken skin
Source: BMC Genomics. 2015 Jan 23;16(1):22. doi: 10.1186/s12864-014-1202-9 (PMC4326372; doi:10.1186/s12864-014-1202-9)
Supplement: Additional file 1: Table S1. — Primer sets for RT-qPCR. Table S2. Primer sets for ChIP-qPCR. Table S3. KEGG pathways identified based on the differentially regulate genes and co-regulated genes from cosine similarity analysis. Table S4. Cosine similarity analysis results, for E7fe vs E9se; E7fm vs E9sm; E9fe vs E11se; E9fm vs E11sm. Figure S1. GSEA coupled with interaction pathway analysis on embryonic development GO terms for the comparisons between feather and scale of matching developmental stages (E7fe vs E9se, E7fm vs E9sm, E9fe vs E11se, and E9fm vs E11sm); as well as for the comparison of same skin component before and after development (E7fe vs E9fe, E7fm vs E9fm, E9fe vs E11fe, and E9fm vs E11fm). Figure S2. RT-qPCR of calcium channel members at the epithelium (biological repeats ≥ 3). Figure S3. ChIP-qPCR of the potential enhancer regions that may be associated with CACNA1H gene activities. Figure S4. ChIP-qPCR of the potential enhancer regions that may be associated with CACNA2D1 gene activities. Figure S5. ChIP-qPCR of the potential enhancer regions that may be associated with CACNA1C gene activities. Figure S6. ChIP-qPCR of the potential enhancer regions that may be associated with CACNA1G gene activities. Figure S7. ChIP-qPCR of the potential enhancer regions that may be associated with CACNA2D3 gene activities. [file 12864_2014_1202_MOESM1_ESM.zip › 8893653601298860_add1.docx]

**Supplementary Tables**

**Supplementary Table 1.** Primer sets for RT-qPCR. Asterik marks are primers for housekeeping genes GAPDH (cytosolic protein control) and β-actin (membrane protein control)

| Primer set | Note | Forward Primer | Reverse Primer |
| --- | --- | --- | --- |
| ATP2B1 |  | 5'-GGAACTTCGAGCTACAGATGC-3' | 5'-CCCAAAAACTGCTTCTCTCCT-3' |
| CACNA1D (1) | α_1_ subunit | 5'-TCATTATTTGGGAGAGCAGGA-3' | 5'-GGGTGATGGTAACCTTTTGG-3' |
| CACNA1D (2) | α_1_ subunit | 5'-CCGTGCCCTTTTCTGTTTAT-3' | 5'-CATCTTCTGGGAATGGGATG-3' |
| CACNA1H (1) | α_1_ subunit | 5'-TGAGACGCCTGGAGAAAAAG-3' | 5'-GGCTGGTTGTAGTGCTCCAT-3' |
| CACNA1H (2) | α_1_ subunit | 5'-CCAGCCACTATCTCGACCTC-3' | 5'-AGGACTGCCTCGAACACAAA-3' |
| CACNA2D1 (1) | α_2_δ subunit | 5'-ACTCCAAGCAGTCCATCATGT-3' | 5'-TGTAATTCCACCATCCGTAACA-3' |
| CACNA2D1 (2) | α_2_δ subunit | 5'-TGAAACCTGCAGTTGTTGGA-3' | 5'-CCCGATTTGACATCAGAAGAA-3' |
| CALM1 |  | 5'-GCTGGGTCAAAATCCAACAG-3' | 5'-CTCGCTGTCTGTGTCCTTCA-3' |
| CAMK1G |  | 5'-CAGGTCCTGACAGCAGTGAA-3' | 5'-GACATGATGCCGTTCTGCT-3' |
| CAMK2D |  | 5'-CTGTCGTCTCTTGAAGCATCC-3' | 5'-CTGGCATCTGCTTCACTGTAA-3' |
| CAMK4 |  | 5'-ATCGACGGCTCTAACAAGGA-3' | 5'-ATGTGAAAGGCGAAGAAGGA-3' |
| GAPDH * |  | 5'-CCTCTCTGGCAAAGTCCAAG-3' | 5'-ACAAGTTTCCCGTTCTCAGC-3' |
| ITPKA |  | 5'-CCAGTGATTAGCCCCTTCAA-3' | 5'-TCAATCGCTCAAAACACTCCT-3' |
| ORAI1 (1) |  | 5'-GTCCTGGCGGAAGCTCTAC-3' | 5'-CGTGGGTAGTCATGTTCTGC-3' |
| ORAI1 (2) |  | 5'-GTATGCATCGGCACATTGAG-3' | 5'-GGTCAAGGGGGTTCTTCTTC-3' |
| PRKCB |  | 5'-CTCACTGCTGTATGGCCTCA-3' | 5'-TCGGCCCTGATGTATATCCT-3' |
| SLC8A3 |  | 5'-CTTTGCGGTGGGAATTATTG-3' | 5'-AGCATGGTCATCATCCAAGA-3' |
| β-actin * |  | 5'-CTCCCTGATGGTCAGGTCAT-3' | 5'-AGACAGCACTGTGTTGGCATA-3' |
| CACNA1C (1) | α_1_ subunit | 5'-AACGCAGAGGAAAACTCTCG-3' | 5'-GAAGAGCCACATAGGGGAGA-3' |
| CACNA1C (2) | α_1_ subunit | 5'-GGGATGCAGGTGTTTGGTAA-3' | 5'-GGGGTCGCACTTCTTGTCT-3' |
| CACNA1S (1) | α_1_ subunit | 5'-TATGTTGCCCTGCTGATCG-3' | 5'-GTGGCACACCTGAAGAGCA-3' |
| CACNA1G (1) | α_1_ subunit | 5'-CCTGCTCTGCTTCTTCGTCT-3' | 5'-AAGGGGTTCTCGTCCTCATT-3' |
| CACNA2D3 (1) | α_2_δ subunit | 5'-GAAAAGAGCCATTGCTCCAA-3' | 5'-CCAAGGAAGGCAACTTCAAC-3' |
| ORAI2 (1) |  | 5'-TGCTCTCTGGATTTGCAATG-3' | 5'-GAATGCAGGTGCTGATGAGA-3' |

**Supplementary Table 2.** Primer sets for ChIP-qPCR. ”US denotes up-stream, “IN” denotes intron, and “DS” demotes down-stream.

| Primer set | Forward Primer | Reverse Primer |
| --- | --- | --- |
| CACNA1D_US-1 | 5'-TGGCTTAGGATGGGAATGG-3' | 5'-CCAGCTCGGCTCTCCAC-3' |
| CACNA1D_IN-1 | 5'-CCACCACACAGTATGGGATTT-3' | 5'-CCTCCTAATGACCAACTCTGCT-3' |
| CACNA1D_IN-2 | 5'-CACTGTCAAATGGGAAACCA-3' | 5'-TTTGGAATCCTAAAGTCACACAG-3' |
| CACNA1D_IN-3 | 5'-GGAGAATGCAGTGCGTGTAA-3' | 5'-GCCTGAAGTTAGGCAACAAAA-3' |
| CACNA1H_US-1 | 5'-AGTGATGGGGAACCACTGAG-3' | 5'-TGCCGATGATTCACTTTTGA-3' |
| CACNA1H_IN-1 | 5'-AGCCACACAAGCTGCCTTT-3' | 5'-CTAGCTGTGTACCTGCCCAAC-3' |
| CACNA2D1_US-2 | 5'-CTGCCACAGAGAAGGGTGTA-3' | 5'-GGGCTGCATTCATATACTCG-3' |
| CACNA2D1_US-1 | 5'-CAAAGGGTTTCTTTTTCTTTGC-3' | 5'-TCAGACGCTTCTCCTGACTTT-3' |
| CACNA2D1_IN-1 | 5'-GCTGCAGCAAATGTATAACG-3' | 5'-AAGAATGGCAAACCAAATGC-3' |
| CACNA2D1_DS-1 | 5'-CAGGCTTTTCAGAAGGGATAGA-3' | 5'-TCCTCCTGATCAGATGTCCTG-3' |
| CACNA1C_US-1 | 5'-CTTCCCTCACACCTCCTCAT-3' | 5'-CCTACATCAGCAGCATCGTG-3' |
| CACNA1C_US-2 | 5'-CAGCTGGAACATCTGGAAGC-3' | 5'-GCTTCTGCTCCTCCTCTTCA-3' |
| CACNA1C_US-3 | 5'-TCCTAAAAACCACCCAACGA-3' | 5'-GAGGGCCAGGTTGACTCAT-3' |
| CACNA1C_DS-1 | 5'-GTGTACCACAGCGCAAAGAA-3' | 5'-TCAGGAGAACGTCTTGAGCA-3' |
| CACNA1C_DS-2 | 5'-AGCGGTTCGTTTCTTGTAGC-3' | 5'-CTCGGAAACAGCAGATGTGA-3' |
| CACNA1G_DS-1 | 5'-TTGGAGCGAGGATTAGTTCTG-3' | 5'-TGAGCACCACAGGAGCAGTA-3' |
| CACNA1G_DS-2 | 5'-TACACACGGTGTTGGCACA-3' | 5'-TTGGAGTCGGATCTGAACCT-3' |
| CACNA1G_IN-3 | 5'-CTGGGAAGATGGATGAGGTG-3' | 5'-GGGAAAGCACAGCTCAGAAT-3' |
| CACNA1G_IN-2 | 5'-GCACAACGACCTGCATTG-3' | 5'-ACGCACCCACAGCTCCT-3' |
| CACNA1G_IN-1 | 5'-CAGCGCTGTCACTCCTCTC-3' | 5'-TCCAAGTCCTTTGCTTGCTT-3' |
| CACNA2D3_US-1 | 5'-CACGTTCAAAGGAGCACGTA-3' | 5'-GATGCTGGGGGTATTTTGTC-3' |
| CACNA2D3_IN-1 | 5'-TGTTTCTTCTGTTGTTTCAGTGG-3' | 5'-CGACATCTAATGAGCAAAATGG-3' |
| CACNA2D3_IN-2 | 5'-CAATGAAATGAGGGCATTGA-3' | 5'-CAGCAGCATTCTTTCCCTTC-3' |

**Supplementary Table 3.** KEGG pathways identified based on the differentially regulate genes and co-regulated genes from cosine similarity analysis. Genes are identified from each feather-scale comparison with similar developmental plasticity. KEGG Pathways are ranked based on the number of comparisons, in which showed high significance (p-value <0.05).

| **KEGG Pathways** | **E7fe vs. E9se** | **E7fm vs. E9sm** | **E9fe vs. E11se** | **E9fm vs. E11sm** |
| --- | --- | --- | --- | --- |
| ECM-receptor interaction | 4.94e-08 | 1.59e-03 | 5.78e-09 | 2.06e-03 |
| Focal adhesion | 3.88e-08 | 1.39e-05 | 1.32e-07 | 6.21e-04 |
| Melanogenesis | 3.34e-05 | 2.89e-02 | 2.29e-07 | 1.46e-02 |
| Calcium signaling pathway | 5.79e-03 | 5.28e-05 | 2.23e-02 | > 0.05 |
| Vascular smooth muscle contraction | > 0.05 | 2.30e-05 | > 0.05 | 7.21e-04 |
| Gap junction | > 0.05 | 1.42e-04 | > 0.05 | 1.66e-02 |
| Arginine and proline metabolism | > 0.05 | 1.95e-03 | > 0.05 | 1.07e-02 |
| Cell adhesion molecules (CAMs) | > 0.05 | 1.01e-02 | 1.45e-03 | > 0.05 |
| Adherens junction | > 0.05 | 6.74e-03 | 4.09e-02 | > 0.05 |
| Wnt signaling pathway | 3.94e-02 | > 0.05 | 1.56e-02 | > 0.05 |

**Supplementary Table 4.** Cosine similarity analysis results, for E7fe vs E9se; E7fm vs E9sm; E9fe vs E11se; E9fm vs E11sm. Non-redundant microarray probes co- or reciprocally regulated with more than 10 seeds were listed. See file: Cosine Similarity Selected Genes.xlsx

**Supplementary Figures**

**Supplementary Figure 1.** GSEA coupled with interaction pathway analysis on embryonic development GO terms for the comparisons between feather and scale of matching developmental stages (E7fe vs E9se, E7fm vs E9sm, E9fe vs E11se, and E9fm vs E11sm); as well as for the comparison of same skin component before and after development (E7fe vs E9fe, E7fm vs E9fm, E9fe vs E11fe, and E9fm vs E11fm). Highlighted network component are found significant in the comparison (denoted in brackets).


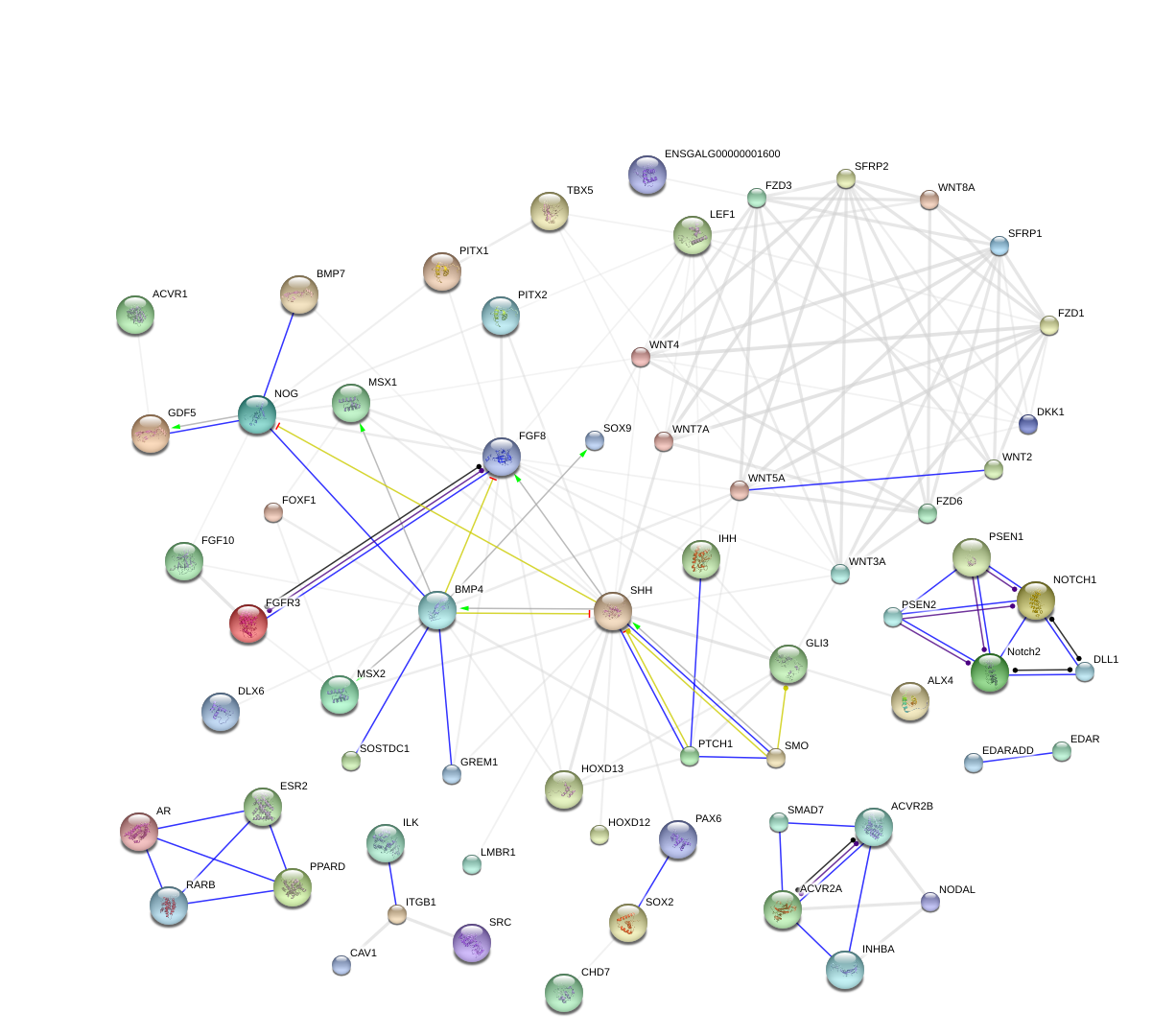


cell fate specification (E7fe < E9fe)

chromatin assembly or disassembly (E7fe > E9sm)

determination of left/right symmetry (E7fe < E9fe; E9sm < E11sm)

embryonic digit morphogenesis (E9sm > E7fm; E11sm > E9fm)

embryonic hindlimb morphogenesis (E9sm < E7fm)

embryonic limb morphogenesis (E9se > E7fe; E9fe > E7fe)

hair follicle development (E11sm < E9sm)

negative regulation of BMP signaling pathway (E9fm > E7fm)

negative regulation of epithelial cell proliferation (E9fm > E7fm; E11sm > E9sm)

positive regulation of canonical Wnt receptor signaling pathway (E9fe < E7fe)

**GO terms**

**Interactions**

Activation

Inhibition

Binding

Phenotype

Catalysis

Post-transl. m

Reaction

Expression

Correlation

**Supplementary Figure 2.** RT-qPCR on calcium channel members

*****

*****

*****

*****

*****

*****

*****

*****

*****

*****

*****

*****

*****

*****

**Supplementary Figure 3.** ChIP-qPCR on the potential enhancer regions that may be associated with the CACNA1H gene activities

**B**

**A**

CACNAN1H_US-1

CACNAN1H_IN-1

**D**

**C**

*****

*****

*****

*****

**Supplementary Figure 4.** ChIP-qPCR on the potential enhancer regions that may be associated with the CACNA2D1 gene activities

**A**

**B**

CACNAN2D1_US-1

CACNAN2D1_DS-1

CACNAN2D1_US-2

CACNAN2D1_IN-1

**C**

**D**

*****

*****

*****

*****

**Supplementary Figure 5.** ChIP-qPCR on the potential enhancer regions that may be associated with the CACNA1C gene activities

**B**

**A**

CACNAN1C_DS-2

CACNAN1C_DS-1

CACNAN1C_US-1

CACNAN1C_US-2

CACNAN1C_US-3

*****

*****

**C**

**D**

*****

*****

*****

*****

*****

*****

**Supplementary Figure 6.** ChIP-qPCR on the potential enhancer regions that may be associated with the CACNA1G gene activities

**A**

**B**

CACNAN1G_IN-1

CACNAN1G_IN-2

CACNAN1G_IN-3

CACNAN1G_DS-1

CACNAN1G_DS-2

*****

*****

**C**

**D**

*****

*****

*****

*****

**Supplementary Figure 7.** ChIP-qPCR on the potential enhancer regions that may be associated with the CACNA2D3 gene activities

**B**

**A**

CACNAN2D3_IN-2

CACNAN2D3_IN-1

CACNAN2D3_US-1

*****

**C**

**D**

*****

*****

*****

*****

*****
